# Immunology in Practice: a modular framework to support Master of Science students conference attendance and engagement

**DOI:** 10.1111/imcb.12814

**Published:** 2024-08-14

**Authors:** Malgorzata Trela, Sophie Rutschmann

**Affiliations:** ^1^ Department of Immunology and Inflammation Imperial College London UK

**Keywords:** Community of Practice, conference, postgraduate education, situated learning

## Abstract

Sharing a passion for the advancement of the discipline, the scientific community provides an authentic environment for new members to acquire the knowledge and develop the professional identity needed for their future careers. Supporting opportunities for higher education students to participate in this community can complement their classroom‐based education and be extremely beneficial to their learning. Situated in the authentic environment of the scientific community, conferences are organized events where professionals meet to advance their discipline, and which have been shown to provide unique learning opportunities for university students. Here we present a modular framework created to support Imperial College London's Master of Science in Immunology students’ attendance at the British Society for Immunology Annual Congress. The module's evaluation indicates an overall students’ satisfaction with the content, organization, teaching, assessment, feedback and community aspects of the framework and draws attention to areas of potential improvements. Furthermore, the data emphasize the importance of preconference preparation, of academic mentoring and discusses the role of peer support. Finally, the data highlight the benefits for students of discovering the true breadth and depth of their discipline, of interacting with members of the community and how these contribute to the development of their professional identity.

## Graphical abstract

Higher education students benefit from attending professional conferences. Here we present a new framework to support our Master's students attending an immunology conference and its evaluation. Challenges and benefits for students are discussed.

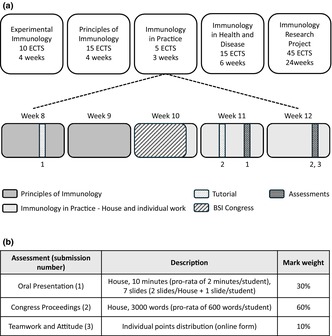

## INTRODUCTION

Scientists are a “group of people informally bound together by shared expertise and passion for a joint enterprise”^1^ (p. 139). They all have the same ambition: to advance knowledge in their discipline. They use specific tools and language to deliver their work and engage in multiple and varied relationships to negotiate their professional activities and contribute to advancing the field. As such, they constitute a Community of Practice (CoP), a concept proposed by Lave and Wenger^2^ in their investigation of apprenticeship learning in which the culture of social participation, nurtured relationships and shared purpose are emphasized. Learning situated in a CoP goes beyond the mere acquisition of factual knowledge and encompasses a progressive mastery of the intricacies of a professional discipline: “The individual learner is not gaining a discrete body of abstract knowledge which (s)he will then transport and reapply in later contexts. Instead, (s)he acquires the skill to perform by actually engaging in the process.”^2^ (p. 14). Moreover, learning situated in a CoP nurtures identity development: “As an aspect of social practice, learning involves the whole person; it implies not only a relation to specific activities, but a relation to social communities – it implies becoming a full participant, a member, a kind of person.”^2^ (p. 53).

Through this legitimate peripheral participation supported by established members of the CoP, newcomers gradually develop an increasing worth of belonging to the CoP and share its enterprise, engagement and repertoire.^2^, ^3^ Consequently, teaching strategies that allow students to participate with others in authentic exchanges and construct new knowledge through becoming a legitimate member of a CoP provide an exciting complementary environment to the traditional classroom, where the emphasis is often mainly on the transmission of factual knowledge and where an individual tutor extracted from the authentic environment of their discipline might not be able to emulate the complexity of engagement, enterprise and repertoire that students will need in their future profession.

Scientific conferences are organized events where academic and nonacademic professionals meet to discuss existing and new knowledge, strengthen established networks, create new ones, generate novel ideas, start collaborations and share job opportunities.^4^, ^5^, ^6^, ^7^ Depending on their position in the community, each delegate will join the conference with a unique agenda.^5^, ^8^ For established members, conferences are a way to not only disseminate the latest work from their research group and hear from collaborators and competitors, but also meet up with long‐term scientific peers and engage in social activities. As places of formal and informal learning, conferences are a popular way for academics to pursue their professional development and strengthen their national and sometimes international visibility.^7^ For newer members of the community, conferences provide an overview of the most up‐to‐date content on the topic and a unique opportunity to socialize and get acquainted with the behaviors, knowledge and skills that are required to develop their identity and allow for their successful entry into a specific professional field.^5^, ^6^, ^8^, ^9^

Several studies have explored the cognitive gain associated with conferences, including that of higher education students (reviewed in Hauss^5^ and Fakunle *et al*.^10^). Conferences are places where the breadth and depth of a discipline are displayed and where knowledge and insights into its trends are gained.^11^, ^12^ Learning takes place through connections made with prior knowledge and professional experience, as well as through conversations and unexpected interactions.^11^, ^12^, ^13^ In addition, attending conferences appears to benefit students’ development of scholarly independence, sense of self, motivation, creativity and professional identity and provides an anchor into a profession, an opportunity to make important career decisions.^5^, ^6^, ^9^, ^10^, ^13^, ^14^, ^15^ Finally, conferences are places where students can also connect with the nonacademic body of the community, opening possibilities for alternative career options to be explored.^5^

While professional conferences provide an exciting, authentic and unique opportunity for students to develop their knowledge, skills and professional identity, attending such an event for the first time or as a very new member of a community can be daunting, intense and sometimes anxiogenic, feelings that can get in the way of the full range of benefits discussed above.^15^, ^16^ To mitigate, several frameworks supporting students’ attendance to professional conferences have been implemented and their positive impact evidenced.^14^, ^15^, ^16^, ^17^ Frameworks in which students plan their conference attendance, establish a strategic agenda and understand the opportunities offered by incidental encounters are helpful in developing a proactive and beneficial consumer stance.^9^, ^18^ In addition, frameworks in which academics mentor students before and after the event have been shown to increase students’ confidence, help understand the community better and support the transition of newer members of the CoP from the periphery to its center.^6^, ^9^, ^14^, ^15^, ^19^

Aiming to enhance Imperial College London's (ICL) Master of Science (MSc) in Immunology classroom teaching with this authentic CoP event, and to encourage our students to be participants rather than just attendees, a modular framework in which they join the British Society for Immunology Conference (BSI Congress) was created. Here, we present the module's structure, its longitudinal evaluation over 4 academic years (AYs), insights from our academic tutors and thoughts for those who might want to implement a similar framework for their students.

## BRITISH SOCIETY FOR IMMUNOLOGY CONGRESS

The BSI Congress takes place in 2 consecutive years, followed by a break during the year of the European Congress of Immunology. Organized in a United Kingdom location over 4 days in the first week of December, it attracts more than 1500 delegates from around the world. Its content is delivered through traditional keynotes and parallel sessions, alongside poster exhibitions. Keynotes are long plenary sessions usually presented by invited leaders in the field. Parallel sessions on specific themes selected by the Congress committee are shorter presentations delivered by group leaders, postdoctoral researchers and sometimes PhD students. Posters are mainly, but not exclusively, presented by students and postdoctoral researchers over two evening's drinks receptions. Poster presentations and parallel sessions talks are selected from a pool of submitted abstracts. In the afternoon before the opening keynote lecture, a “Bright Sparks” session sees presentations from PhD students and postdoctoral researchers compete for prizes. Finally, the Congress is sponsored by scientific companies and societies which display their activities in the poster and breaks hall. In December 2020, at the height of the severe acute respiratory syndrome coronavirus 2 [SARS‐CoV‐2; coronavirus disease 2019 (COVID‐19)] pandemic, the BSI Congress was delivered fully online. Since then, it has been delivered as a hybrid event.

As part of its strategy, the BSI aims to support its members’ career development, notably by providing them opportunities to join networking events and become Society members at a significantly reduced rate. Under this aim, UK MSc students from several institutions have been attending the Congress for many years. To ensure that their engagement with the event is supported and maximized, a module centered on the conference, Immunology in Practice, was created and launched in October 2019 within ICL's MSc in Immunology.

## MSc IN IMMUNOLOGY

**Figure 1:**
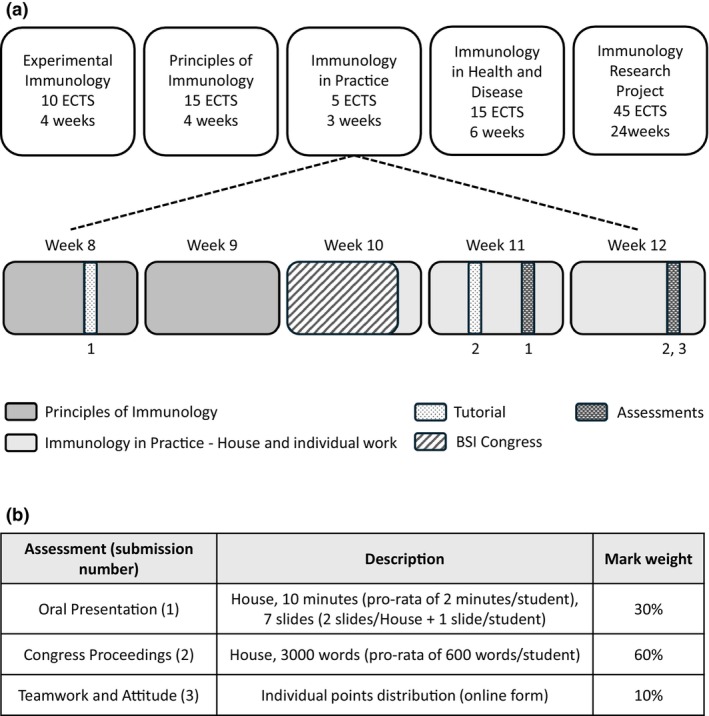
Framework structure and assessments. **(a)** Imperial College London's (ICL) current Master of Science (MSc) in Immunology is delivered through five core modules with sizes ranging from 5 to 45 European Credit Transfer System (ECTS; 1 ECTS = 25 nominal hours of engagement). The ECTS value and module duration are indicated under each module's name (top boxes). The “Immunology in Practice” framework takes place in December, encompassing weeks 10–12 of the academic calendar. The preconference tutorial 1 takes place 7–9 days before the start of the British Society for Immunology (BSI) Congress. During tutorial 2, tutors provide formative feedback to their House on their proceedings draft. The first summative assessment (oral presentation) takes place a week after the conference, followed by the submission of the House's Congress Proceedings and individual teamwork and attitude assessments 2 weeks after the conference. **(b)** Assessments are described for Houses of five students, with guidelines to adapt to different House sizes.

Our framework operates in the following context: ICL's 1‐year full‐time MSc in Immunology hosts between 30 and 37 students per AY. The cohort is, on average, composed of 73% (± 7.3%) female, 27% (± 6.6%) male and one nonbinary student in AY 2022–23, with 51.8% (± 4.9%) international fee status students (Supplementary figure 1). Most students have achieved a 2.1 or first undergraduate degree with an immunology component or hold medical/veterinarian qualifications. Some have professional research experience. All students’ English level meets the International English Language Testing System (IELTS) score of 6.5 overall (6.0 minimum). Since 2019/20, the curriculum has been delivered in five core modules: Experimental Immunology, Principles of Immunology, Immunology in Practice, Immunology in Health and Disease and an Immunology Research Project (Figure 1). The program is delivered with a blend of online asynchronous content and interactive face‐to‐face synchronous sessions.

The program's ambition is to develop graduates with a strong breadth and depth of knowledge in immunology, who can apply the full scientific method, can critically appraise their and others’ experimental data, convincingly communicate scientific ideas and display a successful teamwork attitude. In addition, the program aims to develop graduates who can contextualize the role of the immune system in health and diseases, engage in safe laboratory practices, generate novel experimental data, effectively use scientific evidence to solve problems and think critically.

## IMMUNOLOGY IN PRACTICE

Supporting some of these graduate outcomes, the completion of the “Immunology in Practice” module should allow students to

1. Relate the forefront of immunological topics with core knowledge
2. Identify influencers and predict future research trends in immunology
3. Evaluate and communicate the key aspects of an immunological topic
4. Work effectively as a team to generate an in‐depth coverage of a chosen immunological theme.

Here, “influencers” refer to scientific experts who drive advancement in the discipline.

The module is currently structured as follows (Figure 1): groups of about five students, called Houses, attend the BSI Congress to conduct an in‐depth investigation of an immunological topic of their choice. A preconference session with their academic tutor takes place 7–9 days before the conference. During this tutorial, Houses and tutors examine the program, decide on a topic of investigation, explore the House's strategy including teamwork expectations and discuss the assessments. The 2 weeks following the BSI Congress are entirely free of taught content, except for a second tutorial during which tutors provide feedback on their House's draft proceedings. Throughout the module, students have access to detailed guidelines, marking rubrics and past proceedings.

The first summative assessment takes place a week after the conference ends, with Houses presenting their work orally and answering questions from their peers and assessors (30% of the module's mark). Two weeks after the conference, Houses submit their written Congress Proceedings (60% of the mark). House members write joint introductions and discussions, and each member of the team writes a section on one specific aspect of the overall topic. Finally, a peer assessment of individual contributions to the work (Teamwork and Attitude) is used to moderate the combined proceedings and oral presentation mark and provides the final 10% of the module's mark (adapted from Lejk and Wyvill^20^).

## METHODS

Houses of four to six students, depending on the cohort size, are organized by the program team at the start of the AY, ensuring nationality and gender distribution between the groups.

The MSc program covers the conference registration and Society membership costs. The program also organizes and pays for travel and accommodation for its students.

Students’ module feedback is captured through an individual, anonymous and voluntary Qualtrics survey (Qualtrics, Seattle, WA) accessible online in the Learning Management System. Five themes are explored: organization, content, assessment, feedback and sense of community, using a 5‐point Likert scale: definitely agree, mostly agree, neither agree nor disagree, mostly disagree and definitely disagree. Two free‐text questions complete the survey: “What did you like most about this module?” and “What would you change about this module?”. Students are invited to provide their answers in their own time within 6 weeks after the end of the module. A reminder is sent to all students a week before the closure of the survey.

Tutors’ feedback was captured as part of our periodic program review in an anonymous and voluntary Qualtrics survey in April 2024. Some tutors, but not all, attend the conference. All except one have supported the module since 2020/21, and 6 out of 7 responded to the survey.

Data and free‐text entries were independently analyzed by both authors. The analysis of the free‐text answers identified four themes for each question. Quotes were chosen by the authors to illustrate the main answers for each AY.

Demographic and fee status information originates from administrative records and cannot, therefore, be associated with the evaluation data.

## MODULE EVALUATION: STUDENTS’ PERSPECTIVE

**Figure 2:**
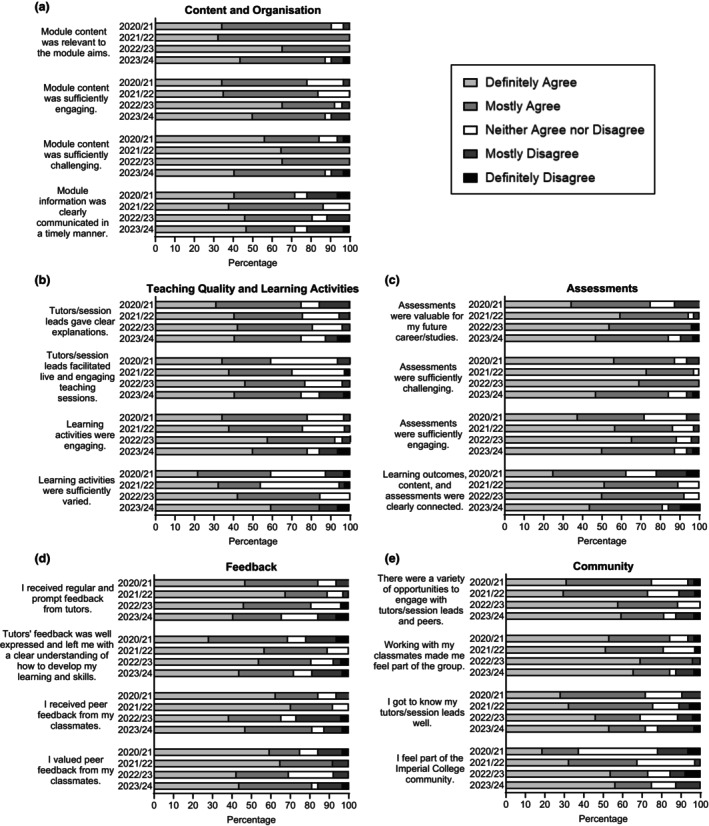
Students’ module evaluation. Every academic year (AY), individual students have the opportunity to fill in an anonymous module feedback survey on the following categories: **(a)** Content and organization, **(b)** Teaching quality and learning activities, **(c)** Assessments, **(d)** Feedback and **(e)** Community. Survey statements and AYs are indicated on the left of the graphs. Five answers are offered for each statement: definitely agree, mostly agree, neither agree nor disagree, mostly disagree and definitely disagree. The survey participation rate was 97% (32 survey responses) in 2020/21, 100% (37 responses) in 2021/22, 87% (26 responses) in 2022/23 and 91% (32 responses) in 2023/24.

Survey participation rates over the four AYs ranged from 87% to 100%, with a total of 127 survey participants. The percentages of respondents in each answer category for each AY are represented in Figure 2. Individual and average values for each answer category across the four AYs, standard deviations and average values for overall positive (definitely agree and mostly agree), neutral (neither agree nor disagree) and negative (mostly disagree and definitely disagree) answers are provided in Supplementary table 1.

- **Content and organization** (Figure 2a). While most students in the individual AY found the content of the module relevant (87.5–100% of positive answers), sufficiently engaging (78.1–92.3%) and sufficiently challenging (84.4–100%), only 71.9–86.5% of the students responded positively in relation to the communication's clarity and timeliness.
- **Teaching quality and learning activities** (Figure 2b). Students found that the session leads gave clear explanations (75–80.8% of positive answers) and that the learning activities were engaging (75.7–92.3%). However, only 59.4–76.9% and 54.1–84.6% of students felt positive about the session leads ability to facilitate live and engaging sessions and about the learning activities being sufficiently varied, respectively. A high and variable level of neutral answers was obtained for this theme (0–40.5%, with an overall average of 17.5%).
- **Assessments** (Figure 2c). Students felt that the assessments were relevant to their future career/studies (75–96.2% of Positive answers), sufficiently challenging (84.4–100%), sufficiently engaging (71.9–88.5%) and they found a clear connection between the learning outcomes, content and assessments (62.5–92.3%). Interestingly, AY 2020/21 felt less positive than the other three years in response to all statements, except to “assessments were sufficiently challenging”.
- **Feedback** (Figure 2d). Most students felt positive about receiving regular and prompt feedback from their tutor (65.6–89.2% of positive answers) and indicated that feedback from their tutor left them with a clear understanding of how to develop their learning and skills (68.8–89.2%). They also felt positive about receiving feedback from their peers (65.4–91.9%) and felt this feedback was valuable (69.2–91.9%).
- **Community** (Figure 2e). Students felt they had a variety of opportunities to engage with session leads (73–88.5% of positive answers) and that working with their classmates made them feel part of the group (81.1–96.2%). However, only 69.2–75.7% of students felt positive about getting to know their session leads well, and 37.5–75% about feeling part of ICL's community. This was the lowest level of positive answers in the survey over the four AYs, with a clear difference between the 2020/21 (37.5%) and the other three AYs (67.6–75%). Finally, a high and variable level of neutral answers was obtained for this “Community” theme (0–40.6%, with an overall average of 14.6%).

Students’ answers to the question “What did you like most about this module?” fall into four themes (Table 1). In all four cohorts, a significant number of students enjoyed the experience of attending and participating in a conference, without necessarily providing further details. Another theme relates to the Congress’ scientific content, with students highlighting its cutting‐edge nature, the variety of fields covered and the discovery of new technologies and their applications. A further theme is linked to the scientific community, including the opportunity for interactions with its members, to observe them at work and to enter an environment that “humanizes science”. The last theme relates to the module itself, with answers highlighting the benefits of teamwork, of socially interacting with classmates, of receiving feedback from the tutors and of developing non‐scientific skills.

**Table 1:** “What did you like most about this module?”: illustrative students’ quotes.

2020/2021	2021/2022	2022/2023	2023/2024
**Attending the conference**			
“The experience of participating in a scientific conference.”	“The experience of attending a conference and getting to attend talks that were of interest to me.”	“Being able to go to the BSI was really engaging and eye opening.”	“I loved the BSI, I thought it was a great way of learning about immunology in practice and will definitely remain as a highlight of my masters at Imperial College. I also liked that we had presentations as part of our assessments so we can learn from our peers.”
**Scientific content**			
“I really enjoyed being able to learn about the new and cutting research being done in the field of immunology, and how that encompassed different fields – it was nice to see the diversity and the implications/applications immunology has on a range of topics which helped me to develop my understanding of the subject.”	“The talks were very informative and provided a great insight into the new work happening.”	“Get a chance to attend to the Congress and to know frontier immunology and technologies from presentations and posters.”	“It was a great opportunity to attend BSI and listen to actual research that is going on in the real world. It gave me an insight what I want to do and what I can do in future.”
**Scientific community**			
“That we were given opportunities to interact with the wider immunologist community.”	“It was a real glimpse of a career in immunology as it showed the field in practice. A great module!”	“I enjoyed the opportunity to attend BSI Congress and get to experience speaking with researchers about their novel insights.”	“The trip to the BSI was a truly enlightening experience going into the world of research seeing first‐hand the names behind all of the publications we read, humanizing the process more.”
**Module organization**			
“The possibility of working as a house group enabled students to create new connections and work as a team towards common goals. Feedback from tutors was highly constructive and based on a suitable balance between positivism and criticism.”	“I really enjoy the congress and teamwork in this module. The assessment of the proceeding is challenging but it helps me to learn how to organize information and how to choose the most important point I wanna [sic] share.”	“Trained my communication skills. I did enjoy the fact that we had meetings with our tutor about the proceedings, it taught me a lot about how to organize my work and how to write better. It also got us closer as a cohort.”	“The fact that we had a chance to get to know everyone from our course and spend quality time together.”

The analysis of free‐text answers identified four themes, presented in no particular order of importance: attending the conference, scientific content, scientific community and module organization. One quote that best illustrates the main answers for each AY is provided.

Interestingly, some students shared how they felt the experience benefited the development of their scholarly independence: “The assessment of the proceeding is challenging but it helps me to learn how to organise information and how to choose the most important point I wanna [sic] share.” (Student 2021/22) and of their professional identity: “Through this module I was able to develop and broaden my understanding of immunology, and also reaffirm why I want to be a scientist.” (Student 2020/21), “This module was an eye‐opener to what my future holds as a scientist, and I feel the tasks completed in this module helped prepare and develop my skills such as note taking, digesting high‐level research and interacting with poster presenters.” (Student 2022/23) and “It was a great opportunity to attend BSI and listen to actual research that is going on in the real world. It gave me an insight what I want to do and what I can do in future.” (Student 2023/24).

When asked “What would you change about this module?”, students’ answers fell into four categories (Table 2). One theme relates to assessment and feedback. Several students suggested that feedback could be more elaborated and detailed, and that additional time could be given to complete the assessments. Only 2 students out of 127 mentioned the assessment getting in the way of enjoying the conference. Another theme relates to teamwork and peer marking, with some students expressing dissatisfaction with marks that are not partly or entirely individual and/or finding the requirement to work in a team and manage specific group dynamics difficult. A further theme links to the immunological topic being investigated: some students were satisfied to be allocated a specific topic while others, in AYs where topics were allocated by the program, would have liked to choose their own avenue of investigation. The final theme is linked to the organization of the module with students commenting on the assessment timings or asking for a whole‐class introduction session to the module.

**Table 2:** “What would you change about this module?”: illustrative students’ quotes.

2020/2021	2021/2022	2022/2023	2023/2024
**Assessment and feedback**			
“The feedback on the individual topics should be more elaborated.”	“More insight into what level of detail is expected for the assignments.”	“I think the assessment took away the main purpose of going to the BSI. I felt like I was more focused on the assessment than trying to network and learn. I couldn’t go to talks that I was genuinely interested in as I had to go to talks for my assessment.”	“More time to do the assignments following the conference and more opportunities for feedback throughout the module.”
**Teamwork and peer marking**			
“I feel that my final mark should be a reflection of my own ability/effort and not that of my colleagues.”	“Working in groups! A few members made me feel left out.”	“I would have preferred a group mark for the intro/discussion, but an individual mark for our own sections. I spent more time editing a teammate's section than I did writing up my own.”	“Perhaps allow for individual proceedings so students can focus on their own topic of interest.”
**Proceedings’ topic**			
“Students should be able to choose their own topics as not all topics may be equally represented in the conferences, and it does not reflect the reality of conferences where participants choose the talks that they are most engaged in.”	“It's difficult to come up with the different topics to write about prior to the actual conference because the abstract of the talks don’t really match up to what was actually presented. Perhaps it might be better to ask for the topics after the conference so students can have a better idea and organization.”	“Make everyone give their topics before BSI to avoid repeating topics.”	“Because the assessment was focused only in one topic it is hard to listen to variety of topics.”
**Module organization**			
“Having all the assessments before the Christmas break would be better rather than have the oral presentation after.”	“It would have been nice to have an introductory session for the module before going to the Congress to go through expectations for the Congress, introduce the assessments and allow students to ask questions.”	“Time scales – perhaps have the presentation after the submission.”	“More time between the Conference and the assignments to consolidate all of the information.”

The analysis of free‐text answers identified four themes, presented in no particular order of importance: assessment and feedback, teamwork and peer marking, proceeding's topic and module organization. One quote that best illustrates the main answers for each AY is provided.

During the COVID‐19 pandemic, the cohorts 2020/21 and 2021/22 attended the conference fully online. Interestingly, this did not seem to have affected the overall level of satisfaction with most surveyed aspects of the module (Figure 2). Some differences are, however, noticeable. A lower level of students in the COVID‐19 AYs definitely agreed with the statement “the module content was relevant to the module aims” (34.4% in 2020/21 and 32.4% in 2021/22) than in the two non‐COVID‐19 AYs (65.4% in 2022/23 and 43.8% in 2023/24). Similarly, fewer students definitely agreed with the statement “the module content was sufficiently engaging” in the COVID‐19 AYs (34.4% in 2020/21 and 35.1% in 2021/22) than in the non‐COVID‐19 AYs (65.4% in 2022/23 and 50% in 2023/24). Finally, to the statement “there were a variety of opportunities to engage with tutors/session leads and peers”, fewer students definitely agreed in the COVID‐19 AYs (31.2% in 2020/21 and 29.8% in 2021/22) than in the non‐COVID‐19 AYs (57.7% in 2022/23 and 59.4% in 2023/24). However, these marked differences are not reflected when looking at the combination of positive answers (Supplementary table 1).

Interestingly, to the statement “learning activities were sufficiently varied”, a marked difference in positive answers is obtained between the two COVID‐19 and non‐COVID‐19 AYs, with 59.4% and 54% of positive answers in 2020/21 and 2021/22 and 84.6% and 84.4% in 2022/23 and 2023/24, respectively.

Few students indicated that they would have preferred to attend the conference in person: “The fact that we didn’t get to attend the conference in real life made the experience less valuable and impactful.” (Student 2020/21). Some students found working entirely online difficult: “It's been difficult to work online all the time … sometimes people doesn’t [sic] engage as much as you expect. I would give recorded conferences to students.” (Student 2020/21) and “Having it online made it a little difficult for me to navigate through the talks” (Student 2021/22). Interestingly, the difficulty of taking notes online has not come up in the years where the conference was attended in person. Other students were quite understanding of the circumstances: “It would have been amazing to be there in person, but unfortunately that was not possible, which is very understandable.” (Student 2021/22), with some bringing up positive aspects of attending an immunology conference during a pandemic: “I think the best this about this module, was that it enabled me to get a unique perspective on the collaborative scientific efforts to control the pandemic. It was refreshing and actually quite moving to see scientists come together and present the efforts of clinicians and researchers from a range of different fields.” (Student 2020/21).

## MODULE EVALUATION: TUTORS’ PERSPECTIVE

In a recent free‐text survey, academic tutors were asked to share their experience of being involved with the framework, including what they felt was most enjoyable, the impact changes made to the module have had on their role and what the benefits and challenges are for their students.

All tutors reported that the highlight of their role was helping their House link the primary content from the conference with the wider literature into one coherent written piece of work: “Seeing how MSc students engage with presentations on primary research findings for the first time and encouraging them to think about what is being shown, why and how it integrates with the literature.” Some of our tutors also attend the Congress with one sharing a wider perspective: “Really nice to see the students ‘wide eyed’ over the breadth and depth of their chosen field of Immunology. It is fantastic to witness how they all seem to grow in confidence during that week and find themselves at home within the community.”

The combination of responses suggests that clear guidelines around the role of the academic tutor, a better‐defined structure around the running of the tutorials and the introduction of a preconference session with their House have all been beneficial developments. These also contribute to a more uniform support across Houses: “It has become a bit more organized with the experience gained and we are more confident in what we are asking the students to do. We also now as tutors have more structured directions in what and how much support we give the students – aiming for all tutor groups to gain the same level of support.”

Tutors feel that students very much benefit from the opportunity to attend a professional conference and to be exposed to the discipline: “I think it is a huge benefit to the students to see ‘the world’ that they are entering. To understand the language of immunology and how complex subjects may be conveyed well to an audience. They get a good feel for the now very big field of immunology.”

From their perspective, the module comes with several challenges for the students in terms of scientific content, of making connections with their prior knowledge and of time management: “The challenge is understanding how to organise their time and cope with the intensity. Students can often also struggle conceptually with content if it doesn’t fit with their foundational knowledge.”. They identify that working as a group can be challenging for some Houses but argue that teamwork is required to cover topics with the necessary depth and breadth: “Group work in this module is necessary as the students need to divide themselves to attend all the talks from a chosen topic.”.

## DISCUSSION

Launched in 2019, the Immunology in Practice module has been the framework supporting ICL's MSc in Immunology students’ attendance at the BSI Congress. Our evaluation indicates that most aspects of the framework have been overall very well received by every cohort of students and by academic tutors over the past 4 AYs. The module undoubtedly comes with its challenges but provides a unique opportunity for students to rub shoulders with our community of immunologists.

Before discussing the module's challenges and benefits, some limitations need to be considered.

The module feedback survey presented here was purposefully designed to allow the evaluation of all taught modules on the MSc program. Although this permits a longitudinal cross‐taught module and longitudinal monitoring of students’ satisfaction, it does not provide the flexibility required to evaluate each module's specific nuances. This, possibly combined with the students rating the conference with the same expectations they have for classroom‐based sessions, might contribute to the higher level of neutral responses for some survey questions (e.g. “tutors gave clear explanations” or “tutors facilitated live sessions in an engaging way”). Additional and bespoke module questionnaires could be circulated to students, but this comes at the risk of negatively impacting response rates.

Furthermore, the very nature of a module with many variables over which the MSc program team has limited control, for example, a worldwide pandemic, changes in Congress venue or some aspects of traveling and accommodation logistics, makes a reliable evaluation of isolated interventions difficult. This is further complicated by the fact that every year sees a new cohort of 1‐year full‐time postgraduate students whose satisfaction can therefore only be expressed without reference to previous versions of the module.

### Framework

The longitudinal assessment of students’ satisfaction over 4 AYs shows an overall high level of satisfaction, above 70%, for all investigated aspects but one. Although the low level of positive answers obtained around feeling part of ICL's community might be related to having the module mainly delivered by an external party, the very low positive answers obtained in 2020/21 might have been additionally impacted by the COVID‐19 pandemic.

The highest average satisfaction rates, above 90%, were obtained in relation to the content's relevance and to the challenge levels posed by the module's content and assessments. For the latter, the academic and social benefits of working as a group, the novelty of the assignments and the fact they allowed students to develop new skills have been cited as positive aspects. Nevertheless, students have also made insightful suggestions on how aspects of the assessments and group work could improve. Their feedback has led to several modifications, including the publication of more detailed assessment guidelines, increased clarity on the peer moderation system, enhanced tutorial guidelines, the addition of a preconference tutorial and clarification on the fact that students are not expected to attend all sessions/posters on their chosen topic, but can also attend talks on other subjects that might be of personal interest to them. Some of these changes might explain the improvement seen between 2020/21 and the subsequent AYs in three of the four assessment survey questions.

The timing of assessments has regularly surfaced in feedback from both students and tutors. With the Congress planning out of our control, the module's organization had to adapt every year. In its first iteration, all assessments took place in January, leading to two main difficulties. Some House members felt compelled to work over the holiday break, creating tensions when this was driven by only some students in the group. This also led to some Houses contacting their tutors over their annual leave. To address these issues, all assessments are now due before the holiday break, resulting in better student and staff experience, increased parity in tutor support between Houses and better alignment with the amount of time students should spend on the smallest module of the program.

Finally, students’ feedback on the group work and marks has always been divided. While some very much enjoy working with others and do not seem to mind having a group mark, some feel that their mark should not be affected by the work of others and suggest receiving an individual mark on their part of the written work, or even conducting the investigation entirely on their own. Although it does come with the need for clear guidelines and expectations, we believe that the system of moderation by peer marking used here benefits teamwork dynamics and supports some students’ engagement with the task.^21^ Unsurprisingly, these challenges are noted by the academic tutors who nevertheless argue that teamwork is required to efficiently cover the sheer quantity of content displayed during the Congress. Finally, attending a conference as a group can act as a safety blanket for some students who might find the experience of operating in a new environment, among a big crowd of mainly strangers, and sometimes with high background noise levels, intimidating and/or anxiogenic.^18^ Finally, others have highlighted the positive impact that peers can have as partners in meaning‐making and as potential future collaborators.^6^

The other aspect of very high satisfaction relates to the module's content level of challenge. Unsurprisingly, the breadth, depth, variety of topics and their wider connections are all highlighted as aspects enjoyed by the students. To help groups navigate this content, the framework now includes a preconference tutorial to discuss the program and help Houses identify the topic they want to investigate. This increased mentoring has been identified as beneficial by our tutors and by others before.^6^, ^9^, ^14^, ^15^, ^16^, ^17^, ^19^ In addition, preplanning and setting a strategy can help students deal with the risk associated with information overload that can lead to discomfort and withdrawal.^18^

### COVID‐19 years

While delegates who have attended in‐person conferences before noticed the differences with the online delivery during the pandemic, many students did not have a previous conference experience to compare with. Although some mention they would have liked the conference to take place in person, this appeared to be more of a wish than highlighting a real issue. Interestingly, both COVID‐19–affected cohorts mentioned the difficulty of taking notes without recordings, while the two more recent cohorts did not. This might result from students being used to having access to recordings for all online taught sessions, an opportunity they did not have with the conference material. This could be further explored to better understand students’ culture around engaging with online and in‐person content.

Four survey answers showed differences between the COVID‐19 and the non‐COVID‐19 AYs. For questions about the relevance of the module content, it being sufficiently engaging and opportunities to engage with tutors/session leads and peers, a difference was observed for the definitely agree but not for the positive answers on the whole. This increase in the most enthusiastic answers might result from a combination of in‐person attendance, enhanced program support and some return to normality after 2 years of the pandemic. Together, these might have tipped the students’ answers from mostly to definitely agree, but not be enough to change the opinion of less enthusiastic students. The fourth difference relates to the variety of learning activities, which most probably directly benefited from in‐person attendance where students had access to the same talks as online attendees, but could also enjoy interactions around posters, with scientific companies’ representatives and other informal networking opportunities.

### Benefits for the students

Although this study focuses on the evaluation of a specific modular framework, it also identifies several benefits for students to attend a professional conference. Closely aligned with the evaluation of other frameworks for undergraduate, MSc and PhD students, our data corroborate professional conferences as opportunities for students to discover the depth of knowledge, values and trends in their discipline.^9^, ^11^, ^12^ In addition, our findings also indicate a positive effect on scholarly independence and motivation but do not particularly highlight an impact on our students’ creativity.^5^, ^6^, ^9^, ^10^, ^11^, ^12^, ^13^, ^14^, ^15^ Finally, our tutors discuss learning through connections with prior knowledge, but associate it with one of the challenges of our framework.^11^, ^12^, ^13^ Importantly, the successful achievement by all Houses since 2019 of the Quality Assurance Agency (QAA) level 7 Module learning outcomes aligns with learning taking place.^22^

CoP is the educational lens that has supported both the development and the longitudinal evaluation of our framework. Although the quantitative satisfaction rates presented here do not assess this aspect of the module, the opportunity to interact with the scientific community in its practice was identified in several aspects of the students’ free‐text positive feedback. Beyond the specific “community” theme, they mention aspects of joint enterprise, notably the generation of new immunology knowledge; mutual engagement, such as the community coming together during the pandemic or being able to put names behind the publications; and shared repertoire, for example, the presentations, posters and technologies. These all concur with conferences supporting the development of a sense of self as described by others, which our students express when they talk positively about the experience, confirming their professional identity and choices.^5^, ^6^, ^9^, ^10^, ^13^, ^14^, ^15^, ^23^

Finally, others have described learning at conferences to take place through unexpected interactions.^11^, ^12^, ^13^ This has not been formally evidenced by our evaluation data, but can be reported by the authors who, as delegates themselves, have anecdotally observed engaged discussions between MSc students and varied members of the CoP during the networking sessions. These unexpected interactions led to very tangible outcomes, with some of our MSc students securing their own research project with immunologists from other institutions met at the Congress.

Taking a class of students to a conference might look daunting but provides a unique opportunity for students to leave the classroom and experience the scientific CoP in one of its most vibrant events. Our experience, combined with the work of others, identifies key aspects that help maximize the benefits for students and for the scientific community itself. As CoP old‐timers, conference organizers, taught program teams and PhD supervisors have a duty to guide newcomers and can reflect on some of the following, non‐exhaustive, suggestions:

1. The level of support required by students seems inversely correlated with their seniority status.^6^, ^9^, ^11^, ^15^ Undergraduate and MSc taught students benefit from scaffolded frameworks, with significant support from academic tutors. Junior PhD students might need guidance from their supervisor to identify a suitable event, submit their research work for poster presentation or attend with a group of peers. Advanced PhD students might benefit from getting more involved with the CoP by, for example, presenting their work in a parallel session and/or organizing parts of the event. They might also benefit from skill‐building workshops around academic writing, research methods or research funding opportunities.
2. Having a conference attendance strategy is key to displaying a consumer approach.^6^, ^9^, ^11^, ^18^ Planning session(s) within the framework or as part of a group meeting ahead of the event will support a more meaningful and beneficial experience. Planning with peers can help socialization, especially for less established or more introvert individuals, but can also get in the way of interacting with unfamiliar members of the community. Including networking events and mentoring schemes in the event can benefit new members.
3. Learning significantly benefits from participation; links made between new and prior knowledge; reflection on experience; and formal, informal and serendipitous interactions.^8^, ^11^, ^12^, ^13^ Learning, therefore, benefits from the conference's topic and the participant's field of inquiry being closely aligned and relevant. It also benefits from the event's program, which allows time and space for delegates to reflect and interact, and from established members sharing their knowledge openly, interactively and constructively.

Finally, CoP needs to engage in a reproductive cycle that allows new members to “contribute, support, and eventually lead the community into the future”^24^ (p. 39). This cycle, essential to the long‐term thriving of the CoP, needs to be proactively supported by established members who need to reflect on how their own journey from peripheral participation to full membership took place and to proactively lower participatory barriers for newcomers.^8^, ^9^ To do so, they can also consider covering their students’ attendance costs, introducing newcomers to their own professional network, exchanging postconference reflections, ensuring a diverse representation in the CoP and proactively connecting students with others to support their next career move. In return, junior scientists bring with them novel approaches and fresh perspectives which challenge established paradigms, foster scientific curiosity, push boundaries and ultimately ensure science's future.

## AUTHOR CONTRIBUTIONS

**Malgorzata Trela:** Formal analysis; writing – original draft. **Sophie Rutschmann:** Conceptualization; data curation; formal analysis; investigation; methodology; writing – original draft.

## CONFLICT OF INTEREST

The authors have no conflict of interest to declare.

## Supporting information


Supplementary Figure 1.
Supplementary Table 1.

## Data Availability

The data that support the findings of this study are available from the corresponding author upon reasonable request.

## References

[1] Wenger EC , Snyder WM . Communities of practice: the organizational frontier. Harv Bus Rev 2000; 78: 139–145.11184968

[2] Lave J , Wenger E . Situated Learning: Legitimate Peripheral Participation. Cambridge: Cambridge University Press; 1991.

[3] Wenger E . Communities of Practice: Learning, Meaning, and Identity. Cambridge: Cambridge University Press; 1999.

[4] Hansen TT , Pedersen DB . The impact of academic events – a literature review. Res Eval 2018; 27: 358–366.

[5] Hauss K . What are the social and scientific benefits of participating at academic conferences? Insights from a survey among doctoral students and postdocs in Germany. Res Eval 2021; 30: 1–12.

[6] Kuzhabekova A , Temerbayeva A . The role of conferences in doctoral student socialization. Stud Grad Postdr Educ 2018; 9: 181–196.

[7] Sanders K , Kraimer ML , Greco L , *et al*. Why academics attend conferences? An extended career self‐management framework. Hum Resour Manage 2022; 32: 100793.

[8] Hilliard TW . Learning at conventions: integrating communities of practice. J Conv Event Tour 2006; 8: 45–68.

[9] Chapman DD , Wiessner CA , Morton J , Fire N , Jones LS , Majekodunmi D . Crossing scholarly divides: barriers and bridges for doctoral students attending scholarly conferences. New Horiz Adult Educ Hum Resour Dev 2009; 23: 6–24.

[10] Fakunle O , Dollinger M , Alla‐Mensah J , Izard B . Academic conferences as learning sites: a multinational comparison of doctoral Students' perspectives and institutional policy. Int J Dr Stud 2019; 14: 479–497.

[11] Campbell A , Wick D , Marcus A , Doll J , Yunuba Hammack A . “I felt like I was not just a student:” examining graduate student learning at academic and professional conferences. Stud Grad Postdr Educ 2021; 12: 321–337.

[12] Cherstrom CA . Making connections: attending professional conferences. Adult Learn 2012; 23: 148–152.

[13] Mabrouk PA . Survey study investigating the significance of conference participation to undergraduate research students. J Chem Educ 2009; 86: 1335.

[14] Hyland N , Kranzow J . Innovative conference curriculum: maximizing learning and professionalism. IJ‐SoTL 2012; 6: 1–15.

[15] Flaherty EA , Urbanek RE , Wood DM , *et al*. A framework for mentoring students attending their first professional conference. Nat Sci Educ 2018; 47: 1–8.

[16] Wood L , Louw I , Zuber‐Skerritt O . Enhancing postgraduate learning and development: a participatory action learning and action research approach through conferences. Action Learn 2017; 14: 120–135.

[17] Fiorentino LH , Manson M , Whalen S . Encouraging students to attend the National Convention. J Phys Educ Recreat Dance 2005; 76: 46–49.

[18] Boucouvalas M . How to profit from attending a conference. New Directions and Continuing Education (NDACE) 1985; 1985: 43–56.

[19] Turner CSV . Lessons from the field: cultivating nurturing environments in higher education. Rev High Educ 2015; 38: 333–358.

[20] Lejk M , Wyvill M . Peer assessment of contributions to a group project: a comparison of holistic and category‐based approaches. Assess Eval High Educ 2001; 26: 61–72.

[21] Shishavan HB , Jalili M . Responding to student feedback: Individualising teamwork scores based on peer assessment. Int J Educ Res Open 2020; 1: 100019.

[22] QAA, Quality, Assurance *et al*. The frameworks for higher education qualifications of UK degree‐awarding bodies. 2024.

[23] Stiwich KD , Ross V . Increasing Students' sense of belonging at research conferences. SPUR [Scholarship and Practice of Undergraduate Research Journal] 2022; 5: 9–15.

[24] Barab SA , Duffy TM . In: Jonassen DH , Land SM , eds. From Practice Fields to Communities of Practice. Mahwah, NJ: Lawrence Erlbaum Associates; 2000.

