# Supplementary material for: Immunology in Practice: a modular framework to support Master of Science students conference attendance and engagement
Source: Immunol Cell Biol. 2024 Aug 14;103(2):114–26. doi: 10.1111/imcb.12814 (PMC11792769; doi:10.1111/imcb.12814)
Supplement: Supplementary file 1 — Supplementary Figure 1. Supplementary Table 1. [file IMCB-103-114-s001.pdf]

(a)

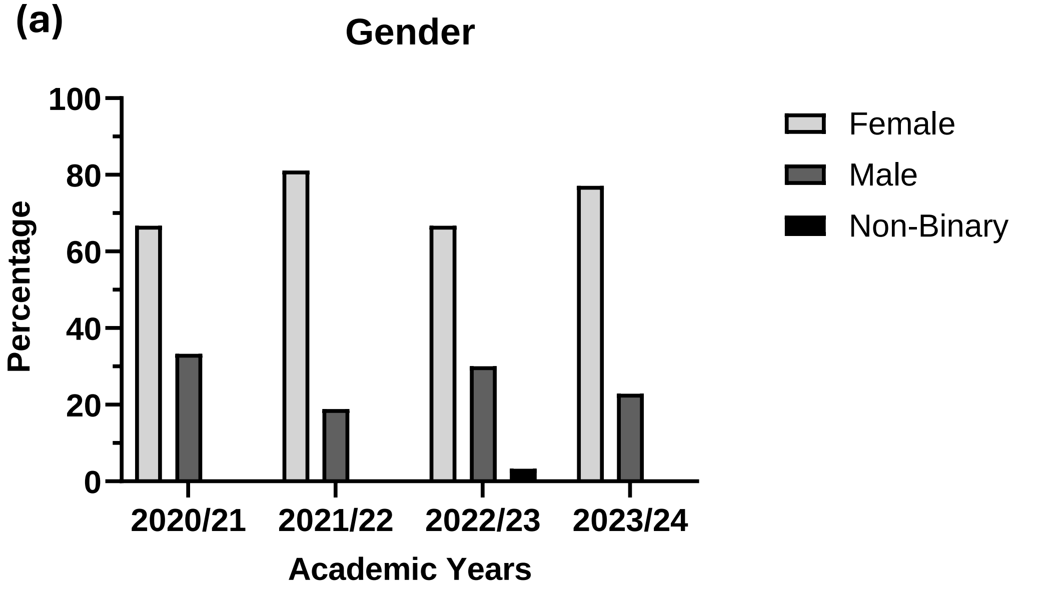

(b)

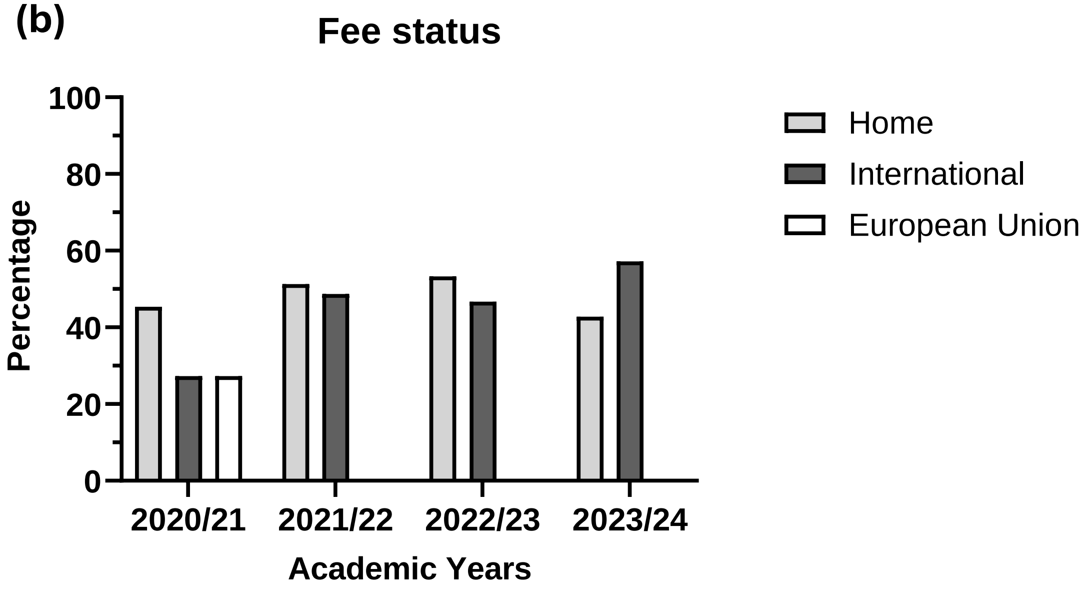

Supplementary Figure 1: Cohorts composition.

(a): Proportion of Female, Male, and Non-Binary students for each Academic cohort.

(b): Proportion of Home, International, and European Union fee status for each Academic cohort. 2020/21 was the last cohort where European students were not paying international student fees.

Supplementary Table 1: Students' evaluation with numerical data, average, variation, and Positive/Neutral/Negative answer categories.

Content and organisation	Definitely Agree	Mostly Agree	Neither Agree nor Disagree	Mostly Disagree	Definitely Disagree
**Module content was relevant to the module aims.**					
2020/21	34.4%	56.3%	6.3%	3.1%	0.0%
2021/22	32.4%	67.6%	0.0%	0.0%	0.0%
2022/23	65.4%	34.6%	0.0%	0.0%	0.0%
2023/24	43.8%	43.8%	3.1%	6.3%	3.1%
**Individual average**	**44.0%**	**50.5%**	**2.3%**	**2.3%**	**0.8%**
Standard Deviation	15.1%	14.4%	3.0%	3.0%	1.6%
Grouped answers	Positive		Neutral	Negative	
**Grouped average**	**94.5%**		**2.3%**	**3.1%**	
**Module content was sufficiently engaging.**					
2020/21	34.4%	43.8%	18.8%	3.1%	0.0%
2021/22	35.1%	48.7%	16.2%	0.0%	0.0%
2022/23	65.4%	26.9%	3.9%	3.9%	0.0%
2023/24	50.0%	37.5%	3.1%	9.4%	0.0%
**Individual average**	**46.2%**	**39.2%**	**10.5%**	**4.1%**	**0.0%**
Standard Deviation	14.7%	9.4%	8.2%	3.9%	0.0%
Grouped answers	Positive		Neutral	Negative	
**Grouped average**	**85.4%**		**10.5%**	**4.1%**	
**Module content was sufficiently challenging.**					
2020/21	56.3%	28.1%	9.4%	3.1%	3.1%
2021/22	64.9%	35.1%	0.0%	0.0%	0.0%
2022/23	65.4%	34.6%	0.0%	0.0%	0.0%
2023/24	40.6%	46.9%	3.1%	6.3%	3.1%
**Individual average**	**56.8%**	**36.2%**	**3.1%**	**2.3%**	**1.6%**
Standard Deviation	11.6%	7.8%	4.4%	3.0%	1.8%
Grouped answers	Positive		Neutral	Negative	
**Grouped average**	**93.0%**		**3.1%**	**3.9%**	
**Module information was clearly communicated in a timely manner.**					
2020/21	40.6%	31.3%	6.3%	15.6%	6.3%
2021/22	37.8%	48.7%	13.5%	0.0%	0.0%
2022/23	46.2%	34.6%	7.7%	11.5%	0.0%
2023/24	46.9%	25.0%	6.3%	18.8%	3.1%
**Individual average**	**42.9%**	**34.9%**	**8.4%**	**11.5%**	**2.3%**
Standard Deviation	4.4%	10.0%	3.5%	8.2%	3.0%
Grouped answers	Positive		Neutral	Negative	
**Grouped average**	**77.8%**		**8.4%**	**13.8%**	

**Individual average** = Average for each answer over 4 Academic Years
**Grouped average Positive =** Sum of Definitely Agree and Mostly Agree averages
**Grouped average Neutral** = Neither Agree nor Disagree average
**Grouped average Negative** = Sum of Mostly Disagree and Definitely Disagree averages

Teaching quality & learning activities	Definitely Agree	Mostly Agree	Neither Agree nor Disagree	Mostly Disagree	Definitely Disagree
**Tutors/session leads gave clear explanations.**					
2020/21	31.3%	43.8%	9.4%	15.6%	0.0%
2021/22	40.5%	35.1%	18.9%	5.4%	0.0%
2022/23	42.3%	38.5%	15.4%	3.9%	0.0%
2023/24	40.6%	34.4%	12.5%	6.3%	6.3%
**Individual average**	**38.7%**	**37.9%**	**14.0%**	**7.8%**	**1.6%**
Standard Deviation	5.0%	4.3%	4.1%	5.3%	3.1%
Grouped answers	Positive		Neutral	Negative	
**Grouped average**	**76.6%**		**14.0%**	**9.3%**	
**Tutors/session leads facilitated live and engaging teaching sessions.**					
2020/21	34.4%	25.0%	34.4%	6.3%	0.0%
2021/22	37.8%	32.4%	27.0%	0.0%	2.7%
2022/23	46.2%	30.8%	19.2%	3.9%	0.0%
2023/24	40.6%	34.4%	9.4%	12.5%	3.1%
**Individual average**	**39.8%**	**30.6%**	**22.5%**	**5.7%**	**1.5%**
Standard Deviation	5.0%	4.0%	10.7%	5.2%	1.7%
Grouped answers	Positive		Neutral	Negative	
**Grouped average**	**70.4%**		**22.5%**	**7.1%**	
**Learning activities were engaging.**					
2020/21	34.4%	43.8%	18.8%	3.1%	0.0%
2021/22	37.8%	37.8%	21.6%	2.7%	0.0%
2022/23	57.7%	34.6%	3.9%	3.9%	0.0%
2023/24	50.0%	28.1%	6.3%	9.4%	6.3%
**Individual average**	**45.0%**	**36.1%**	**12.6%**	**4.8%**	**1.6%**
Standard Deviation	10.8%	6.5%	8.9%	3.1%	3.1%
Grouped answers	Positive		Neutral	Negative	
**Grouped average**	**81.1%**		**12.6%**	**6.3%**	
**Learning activities were sufficiently varied.**					
2020/21	21.9%	37.5%	28.1%	9.4%	3.1%
2021/22	32.4%	21.6%	40.5%	2.7%	2.7%
2022/23	42.3%	42.3%	15.4%	0.0%	0.0%
2023/24	59.4%	25.0%	0.0%	9.4%	6.3%
**Individual average**	**39.0%**	**31.6%**	**21.0%**	**5.4%**	**3.0%**
Standard Deviation	15.9%	9.9%	17.4%	4.8%	2.6%
Grouped answers	Positive		Neutral	Negative	
**Grouped average**	**70.6%**		**21.0%**	**8.4%**	

Assessments	Definitely Agree	Mostly Agree	Neither Agree nor Disagree	Mostly Disagree	Definitely Disagree
**Assessments were valuable for my future career/studies.**					
2020/21	34.4%	40.6%	12.5%	12.5%	0.0%
2021/22	59.5%	35.1%	2.7%	2.7%	0.0%
2022/23	53.9%	42.3%	0.0%	0.0%	3.9%
2023/24	46.9%	37.5%	6.3%	6.3%	3.1%
**Individual average**	**48.6%**	**38.9%**	**5.4%**	**5.4%**	**1.7%**
Standard Deviation	10.8%	3.2%	5.4%	5.4%	2.0%
Grouped answers	Positive		Neutral	Negative	
**Grouped average**	**87.5%**		**5.4%**	**7.1%**	
**Assessments were sufficiently challenging.**					
2020/21	56.3%	31.3%	6.3%	6.3%	0.0%
2021/22	73.0%	24.3%	2.7%	0.0%	0.0%
2022/23	69.2%	30.8%	0.0%	0.0%	0.0%
2023/24	46.9%	37.5%	9.4%	3.1%	3.1%
**Individual average**	**61.3%**	**31.0%**	**4.6%**	**2.3%**	**0.8%**
Standard Deviation	12.0%	5.4%	4.1%	3.0%	1.6%
Grouped answers	Positive		Neutral	Negative	
**Grouped average**	**92.3%**		**4.6%**	**3.1%**	
**Assessments were sufficiently engaging.**					
2020/21	37.5%	34.4%	21.9%	6.3%	0.0%
2021/22	56.8%	29.7%	10.8%	2.7%	0.0%
2022/23	65.4%	23.1%	7.7%	3.9%	0.0%
2023/24	50.0%	37.5%	6.3%	3.1%	3.1%
**Individual average**	**52.4%**	**31.2%**	**11.7%**	**4.0%**	**0.8%**
Standard Deviation	11.8%	6.3%	7.1%	1.6%	1.6%
Grouped answers	Positive		Neutral	Negative	
**Grouped average**	**83.6%**		**11.7%**	**4.8%**	
**Learning outcomes, content, and assessment were clearly connected.**					
2020/21	25.0%	37.5%	15.6%	15.6%	6.3%
2021/22	51.4%	37.8%	10.8%	0.0%	0.0%
2022/23	50.0%	42.3%	7.7%	0.0%	0.0%
2023/24	43.8%	37.5%	3.1%	6.3%	9.4%
**Individual average**	**42.5%**	**38.8%**	**9.3%**	**5.5%**	**3.9%**
Standard Deviation	12.1%	2.4%	5.3%	7.4%	4.7%
Grouped answers	Positive		Neutral	Negative	
**Grouped average**	**81.3%**		**9.3%**	**9.4%**	

Feedback	Definitely Agree	Mostly Agree	Neither Agree nor Disagree	Mostly Disagree	Definitely Disagree
**I received regular and prompt feedback from tutors.**					
2020/21	46.9%	37.5%	9.4%	6.3%	0.0%
2021/22	67.6%	21.6%	8.1%	2.7%	0.0%
2022/23	46.2%	34.6%	15.4%	0.0%	3.9%
2023/24	40.6%	25.0%	18.8%	9.4%	6.3%
**Individual average**	**50.3%**	**29.7%**	**12.9%**	**4.6%**	**2.5%**
Standard Deviation	11.8%	7.6%	5.0%	4.1%	3.1%
Grouped answers	Positive		Neutral	Negative	
**Grouped average**	**80.0%**		**12.9%**	**7.1%**	
**Feedback from tutors was well expressed and left me with a clear understanding of how to develop my learning and skills.**					
2020/21	28.1%	40.6%	9.4%	15.6%	6.3%
2021/22	56.8%	32.4%	10.8%	0.0%	0.0%
2022/23	53.9%	26.9%	11.5%	3.9%	3.9%
2023/24	43.8%	28.1%	9.4%	15.6%	3.1%
**Individual average**	**45.6%**	**32.0%**	**10.3%**	**8.8%**	**3.3%**
Standard Deviation	12.9%	6.2%	1.1%	8.1%	2.6%
Grouped answers	Positive		Neutral	Negative	
**Grouped average**	**77.7%**		**10.3%**	**12.1%**	
**I received peer feedback from my classmates.**					
2020/21	62.5%	21.9%	9.4%	6.3%	0.0%
2021/22	70.3%	21.6%	8.1%	0.0%	0.0%
2022/23	38.5%	26.9%	7.7%	23.1%	3.9%
2023/24	46.9%	34.4%	6.3%	9.4%	3.1%
**Individual average**	**54.5%**	**26.2%**	**7.9%**	**9.7%**	**1.7%**
Standard Deviation	14.5%	6.0%	1.3%	9.7%	2.0%
Grouped answers	Positive		Neutral	Negative	
**Grouped average**	**80.7%**		**7.9%**	**11.4%**	
**I valued peer feedback from my classmates.**					
2020/21	59.4%	15.6%	9.4%	12.5%	3.1%
2021/22	64.9%	27.0%	0.0%	8.1%	0.0%
2022/23	42.3%	26.9%	23.1%	7.7%	0.0%
2023/24	43.8%	37.5%	3.1%	12.5%	3.1%
**Individual average**	**52.6%**	**26.8%**	**8.9%**	**10.2%**	**1.6%**
Standard Deviation	11.3%	8.9%	10.2%	2.7%	1.8%
Grouped answers	Positive		Neutral	Negative	
**Grouped average**	**79.3%**		**8.9%**	**11.8%**	

Community	Definitely Agree	Mostly Agree	Neither Agree nor Disagree	Mostly Disagree	Definitely Disagree
**There were a variety of opportunities to engage with tutors/session leads and peers.**					
2020/21	31.3%	43.8%	18.8%	3.1%	3.1%
2021/22	29.7%	43.2%	16.2%	8.1%	2.7%
2022/23	57.7%	30.8%	11.5%	0.0%	0.0%
2023/24	59.4%	21.9%	6.3%	9.4%	3.1%
**Individual average**	**44.5%**	**34.9%**	**13.2%**	**5.2%**	**2.2%**
Standard Deviation	16.2%	10.6%	5.5%	4.4%	1.5%
Grouped answers	Positive		Neutral	Negative	
**Grouped average**	**79.4%**		**13.2%**	**7.4%**	
**Working with my classmates made me feel part of the group.**					
2020/21	53.1%	31.3%	9.4%	3.1%	3.1%
2021/22	51.4%	29.7%	16.2%	0.0%	2.7%
2022/23	69.2%	26.9%	0.0%	3.9%	0.0%
2023/24	65.6%	18.8%	3.1%	9.4%	3.1%
**Individual average**	**59.8%**	**26.7%**	**7.2%**	**4.1%**	**2.2%**
Standard Deviation	8.9%	5.6%	7.2%	3.9%	1.5%
Grouped answers	Positive		Neutral	Negative	
**Grouped average**	**86.5%**		**7.2%**	**6.3%**	
**I got to know my tutors/session leads well.**					
2020/21	28.1%	43.8%	18.8%	9.4%	0.0%
2021/22	32.4%	43.2%	13.5%	5.4%	5.4%
2022/23	46.2%	23.1%	19.2%	7.7%	3.9%
2023/24	53.1%	18.8%	6.3%	18.8%	3.1%
**Individual average**	**40.0%**	**32.2%**	**14.4%**	**10.3%**	**3.1%**
Standard Deviation	11.7%	13.2%	6.0%	5.9%	2.3%
Grouped answers	Positive		Neutral	Negative	
**Grouped average**	**72.2%**		**14.4%**	**13.4%**	
**I feel part of the Imperial College community.**					
2020/21	18.8%	18.8%	40.6%	15.6%	6.3%
2021/22	32.4%	35.1%	29.7%	2.7%	0.0%
2022/23	53.9%	19.2%	11.5%	7.7%	7.7%
2023/24	56.3%	18.8%	12.5%	12.5%	0.0%
**Individual average**	**40.3%**	**23.0%**	**23.6%**	**9.6%**	**3.5%**
Standard Deviation	17.9%	8.1%	14.1%	5.7%	4.1%
Grouped answers	Positive		Neutral	Negative	
**Grouped average**	**63.3%**		**23.6%**	**13.1%**	
